# Axonal response of mitochondria to demyelination and complex IV activity within demyelinated axons in experimental models of multiple sclerosis

**DOI:** 10.1111/nan.12851

**Published:** 2022-10-07

**Authors:** Simon Licht‐Mayer, Graham R. Campbell, Arpan R. Mehta, Katie McGill, Alex Symonds, Sarah Al‐Azki, Gareth Pryce, Stephanie Zandee, Chao Zhao, Markus Kipp, Kenneth J. Smith, David Baker, Daniel Altmann, Stephen M. Anderton, Yolanda S. Kap, Jon D. Laman, Bert A. 't Hart, Moses Rodriguez, Robin J. M. Franklin, Siddharthan Chandran, Hans Lassmann, Bruce D. Trapp, Don J. Mahad

**Affiliations:** ^1^ Centre for Clinical Brain Sciences University of Edinburgh Edinburgh UK; ^2^ UK Dementia Research Institute University of Edinburgh Edinburgh UK; ^3^ Blizard Institute, Barts and The London School of Medicine and Dentistry Queen Mary University of London London UK; ^4^ Centre for Inflammation Research University of Edinburgh Edinburgh UK; ^5^ Wellcome Trust‐MRC Cambridge Stem Cell Institute, Jeffrey Cheah Biomedical Centre University of Cambridge, Cambridge Biomedical Campus Cambridge UK; ^6^ Institute of Anatomy Rostock University Medical Center Rostock Germany; ^7^ Department of Neuroinflammation, The UCL Queen Square Institute of Neurology University College London London UK; ^8^ Faculty of Medicine, Department of Medicine Hammersmith Campus London UK; ^9^ Department of Immunobiology Biomedical Primate Research Centre Rijswijk The Netherlands; ^10^ Department Pathology and Medical Biology and MS Center Noord Nederland (MSCNN) University Groningen, University Medical Center Groningen Groningen The Netherlands; ^11^ Department Anatomy and Neuroscience Amsterdam University Medical Center (VUMC) Amsterdam Netherlands; ^12^ Department of Neurology and Immunology Mayo College of Medicine and Science Rochester Minnesota USA; ^13^ Department of Neuroimmunology, Center for Brain Research Medical University Vienna Vienna Austria; ^14^ Department of Neuroscience Lerner Research Institute, Cleveland Clinic Cleveland Ohio USA

**Keywords:** axon injury, complex IV, experimental demyelination, mitochondria, multiple sclerosis and neuroprotection

## Abstract

**Aims:**

Axonal injury in multiple sclerosis (MS) and experimental models is most frequently detected in acutely demyelinating lesions. We recently reported a compensatory neuronal response, where mitochondria move to the acutely demyelinated axon and increase the mitochondrial content following lysolecithin‐induced demyelination. We termed this homeostatic phenomenon, which is also evident in MS, the axonal response of mitochondria to demyelination (ARMD). The aim of this study is to determine whether ARMD is consistently evident in experimental demyelination and how its perturbation relates to axonal injury.

**Methods:**

In the present study, we assessed axonal mitochondrial content as well as axonal mitochondrial respiratory chain complex IV activity (cytochrome *c* oxidase or COX) of axons and related these to axonal injury in nine different experimental disease models. We used immunofluorescent histochemistry as well as sequential COX histochemistry followed by immunofluorescent labelling of mitochondria and axons.

**Results:**

We found ARMD a consistent and robust phenomenon in all experimental disease models. The increase in mitochondrial content within demyelinated axons, however, was not always accompanied by a proportionate increase in complex IV activity, particularly in highly inflammatory models such as experimental autoimmune encephalomyelitis (EAE). Axonal complex IV activity inversely correlated with the extent of axonal injury in experimental disease models.

**Conclusions:**

Our findings indicate that ARMD is a consistent and prominent feature and emphasise the importance of complex IV activity in the context of ARMD, especially in autoimmune inflammatory demyelination, paving the way for the development of novel neuroprotective therapies.

## Graphical abstract

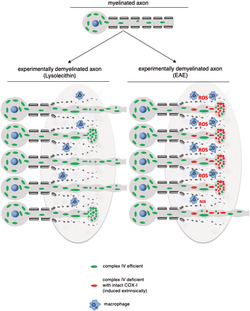

**Key Points**

- The mitochondrial content of demyelinated axons in animal models is significantly greater than myelinated axons, irrespective of the mode of experimental demyelination.
- The increased axonal mitochondrial content following demyelination is consistent with the recently reported axonal response of mitochondria to demyelination (ARMD), which can be enhanced to protect acutely demyelinated axons.
- In lysolecithin‐induced focal lesions, the increased mitochondrial content of demyelinated axons is reflected at the level of complex IV activity, whereas highly inflammatory models such as experimental autoimmune encephalomyelitis (EAE) show a relative lack of complex IV activity within demyelinated axons.
- Complex IV activity of demyelinated axons inversely correlates with the extent of axonal injury in animal models.

## INTRODUCTION

Axonal loss is a cardinal neuropathological feature of multiple sclerosis (MS) [1, 2]. Axonal injury is most prominently observed in actively demyelinating regions of MS, and a growing body of evidence implicates a state of energy failure in the degeneration of demyelinated axons [3]. The bioenergetic need (in terms of ATP) of demyelinated axons is thought to be increased, due to the redistribution of ion channels [4]. In keeping with this increase in energy demand of demyelinated axons, we recently identified a neuronal compensatory mechanism where mitochondria move from the cell body to the acutely demyelinated axon, increasing the axonal mitochondrial content and therefore the energy‐producing capacity [5]. We termed this homeostatic mechanism the axonal response of mitochondria to demyelination (ARMD) [5].

Increased axonal mitochondrial content, reflecting ARMD, has been reported in MS and in a limited number of experimental disease models [6, 7, 8]. In MS, the increase in mitochondrial content of nondegenerated demyelinated axons was accompanied by an increase in activity of mitochondrial respiratory chain complex IV (cytochrome *c* oxidase or COX), where oxygen is reduced to generate ATP aerobically [9, 10, 11]. At the edge of chronic active MS lesions, where acute axonal injury is most prominent, axonal complex IV activity inversely correlated with the extent of inflammation [9]. In highly inflammatory demyelinating environments, such as experimental autoimmune encephalomyelitis (EAE), axonal mitochondrial function is compromised, perhaps by nitric oxide, an inhibitor of complex IV, and mitochondria may be damaged by post‐translational modification of respiratory chain complex subunits due to nitration [12, 13]. Furthermore, axonal mitochondrial transport may be perturbed by inflammation, as evident in EAE [14, 15]. However, it is not known whether ARMD is consistently evident in experimental disease models, whether it always leads to a corresponding increase in mitochondrial respiratory chain complex IV activity in demyelinated axons, nor how any differences in ARMD might relate to axon degeneration.

Against this background, we used nine experimental disease models relevant to MS (highly inflammatory models and non‐autoimmune models) and quantified the mitochondrial content, as well as mitochondrial respiratory chain complex IV activity of both myelinated and demyelinated axons. Complex IV is made up of multiple subunits encoded by both nuclear DNA and mitochondrial DNA. Complex IV deficiency may be due to the loss of subunits, following mitochondrial DNA mutations, and/or modification of subunits by reactive oxygen species in demyelinating regions. Therefore, we assessed complex IV subunit‐I (COX‐I), which is encoded by mitochondrial DNA and forms a key part of the catalytic core of complex IV, in axonal mitochondria to gain insight into the potential cause(s) of complex IV deficiency in axons. Finally, we correlated complex IV activity with axonal injury in demyelinating lesions. We found ARMD to be a consistent feature of all nine animal models. However, axonal complex IV activity was only increased significantly following lysolecithin‐induced demyelination. In all experimental disease models, COX‐I was intact in complex IV deficient axonal mitochondria indicating that the complex IV deficiency in animal models is not due to mtDNA deletions, which leads to the loss of this subunit. The inverse correlation between complex IV activity within demyelinated axons and the extent of axon degeneration suggests a crucial role for complex IV activity in meeting the increased energy demand of the acutely demyelinated axon. This study advances our previous characterisation of ARMD, by including axonal complex IV activity in multiple animal models (highly inflammatory models, such as EAE, and non‐autoimmune models), and highlights the need to protect complex IV activity of mitochondria in demyelinated axons for neuroprotection.

## METHODS

### Experimental disease models

Snap frozen mouse, rat and marmoset CNS tissue of nine disease models and an equal number of age‐matched controls (except for marmoset EAE), were generated, as indicated in Table 1. Tissue from peak demyelination time points, indicated in Table 1, was used in this study. National laws on the principles of laboratory animal care were followed and institutional ethical review committee approvals were obtained for all studies.

**TABLE 1:** Features of the experimental disease models

Model	Species (strain)	Analysis time point (in days)	*n* = (brain, spinal cord)
Focal demyelinating dorsal funiculus lesion:			
LPC (1%) [27]	Mouse (C57BL/6)	5^a^	0, 6
LPS (200 ng) [28]	Rat (Sprague–Dawley)	7^a^	0, 6
Cuprizone‐mediated demyelination of the brain [29]	Mouse (C57BL/6)	42^a^	6, 0
TMEV‐induced inflammatory demyelination [7]	Mouse (SJL/J)	41 (demyelinating)	3, 3
T‐reg depleted active EAE with MOG_35–55_ [30]	Mouse (C57BL/6)	13 (acute)^a^	0, 10
humanised TCR transgenic with spontaneous EAE [31]	Mouse (C57BL/6)	120–150^a^ ^,^ (with clinical scores 1–2 and >3)	3, 3
chronic EAE with subcutaneous spinal cord homogenate [32]	Mouse (Biozzi ABH)	18 (acute)^a^	3, 3
acute EAE with rMOG [33]	Rat (Dark Agouti, Harlan)	14^a^	3, 3
EAE with rMOG_34–56_ [34]	Marmoset (*Callithrix jacchus)*	11 days^a^, on average, post‐EAE score of 2.5	9, 5

^a^ Peak clinical disease or peak demyelination time point for the analysis of axonal mitochondrial parameters. All the time‐points stated above were included in the detection for respiratory‐deficient cells. EAE: experimental autoimmune encephalomyelitis. LPC: lysolecithin. LPS: lipopolysaccharide. MOG: myelin oligodendrocyte glycoprotein. TCR: T‐cell receptor. TMEV: Theiler's murine encephalomyelitis virus. *n* = number of animals used for brain and spinal cord analysis. Equal numbers of age‐matched controls were used, except for marmoset EAE, where 4 age‐matched naïve controls were used.

### Triple label immunofluorescence histochemistry

Cryosections (15 μm thickness) were prepared from the entire snap frozen brain (for sagittal) and alternative 5 mm blocks of the whole spinal cord (for longitudinal sectioning). Cryosections, selected based on the presence of inflammatory infiltrates in haematoxylin/eosin staining in adjacent sections, were air dried for 60 min and fixed in cold 4% paraformaldehyde (PFA) before antigen retrieval was carried out using boiling ethylenediaminetetraacetic acid (EDTA) pH 8.0 for 5 min. The fixed cryosections were then washed and normal goat serum solution (1%) was applied for 30 min at room temperature for blocking. Primary antibodies against either neurofilament, myelin basic protein (MBP) and porin (for mitochondrial content) or neurofilament, complex IV subunit‐I and complex II 70 kDa (for subunit analysis) were applied in TBS for 90 min at room temperature. Appropriate isotype‐specific secondary antibodies (Life Technologies) that are directly conjugated with selected fluorochromes (FITC, rhodamine and Cy5) were applied following three washing steps thereafter. The sections were then washed and mounted using Vectashield with DAPI and stored at −20°C until required for confocal microscopy.

### Sequential complex IV histochemistry and triple label immunofluorescence histochemistry

#### Axonal complex IV activity and complex IV subunit‐I

Complex IV histochemistry was combined with triple immunofluorescent histochemistry with antibodies against neurofilament, complex IV subunit‐I and complex II 70 kDa in order to assess (1) complex IV activity in single axons and (2) identify complex IV‐deficient axonal mitochondria and then assess the complex IV subunit‐I status of mitochondria, which lack complex IV activity. This sequential technique, which is performed in the same tissue section and has already been described and validated in previous publications, is based on the observation that only the mitochondria that lack complex IV activity are immunofluorescently labelled following complex IV histochemistry, because the brown deposit of complex IV histochemical reaction in complex IV active mitochondria prevents the immunolabelling of the mitochondrial respiratory chain complex subunits [9, 16]. Complex IV media consisted of 100 μM cytochrome c, 4 mM diaminobenzidine tetrahydrochloride and 20 μg/ml catalase in 0.1 M phosphate buffer pH 7.0. Cryosections were incubated at 37°C for 30 min and washed in PBS. Cryosections were then proceeded through the triple label immunofluorescent histochemistry steps, as stated above. Secondary antibodies were as follows: anti‐mouse IgG2a Rhodamine‐X Red, Life Technologies; anti‐mouse IgG1 633, Life Technologies, and anti‐chicken 488. The sequentially stained sections were mounted in glycerol with Hoechst nuclear stain and stored at −20°C until required for imaging by the Zeiss Imager Z1 Apotome 2 microscope.

#### Complex IV activity in degenerated axons

Complex IV activity was also investigated in degenerated axons, identified by immunofluorescent labelling of amyloid precursor protein (APP) and synaptophysin. Here, sequential complex IV histochemistry was used with single immunofluorescent histochemistry for APP and synaptophysin in serial sections. The secondary antibody used was anti‐IgG1 rhodamine, Jackson Immunoresearch.

### Microscopy

#### Triple immunofluorescent histochemistry

Confocal microscopy was used to capture fluorescence, using a Zeiss LSM 710. Images were taken using an oil‐lens x63 magnification. Individual optical sections (1 μm thickness) were combined into a Z‐stack in order to track axons longitudinally in the 15 μm thick sections. FITC, TRIC and Cy5 channels were imaged sequentially with the offset and gain kept consistent between sections. Images using SCoRe, a label‐free method to detect myelin based on its reflective properties when excited at different wavelengths, were used to ensure confirmation of myelinated and demyelinated axons when antibodies against myelin were not used in the triple staining protocol [17].

#### Sequential complex IV histochemistry and immunofluorescent histochemistry

brightfield images of complex IV activity and immunofluorescent labelling of axons as well as mitochondria that lack complex IV activity, due to the blocking of immunolabelling by the deposits of complex IV histochemical reaction, were obtained using the Apotome microscope.

In adjacent sections, complex IV activity and immunofluorescent labelling of APP and synaptophysin were similarly captured. Images were taken using a x63 oil lens and brightfield and FITC, TRIC and Cy5 channels were imaged sequentially.

### Quantification

#### Triple immunofluorescence histochemistry

Maximum projection images were obtained using the Zeiss Zen 2.3 software from the confocal Z‐ stacks that contained the axons of interest. For the analysis of mitochondrial content in axons, the presence of NF‐H (at least 50 μm long segment without a terminal ovoid or fragmentation) and lack of MBP in hypercellular areas (DAPI positive) were deemed to be intact demyelinated axons. In order to accurately determine axonal mitochondrial numbers, mitochondria identified by porin labelling that did not co‐localise with NF‐H positive outline were considered to be non‐axonal and cleared from the image using Photoshop software. Individual axons were then cropped on Image J software and a mask was made of individual axonal porin puncta. The axonal mitochondrial content was determined as the total area of porin labelled puncta within a single axon as a percentage of the axon area; 20 axons (at least 25 μm in length) per region were randomly selected from each animal for quantitation and the mean values are indicated in Table 2. The extent of complex IV subunit‐I labelling in all axonal mitochondria was determined as the total area of subunit labelled puncta within a single axon as a percentage of the axonal area (Table 3 column three). Assessors were blinded by coding the axons in ascending numerical order.

**TABLE 2:** Changes in axonal mitochondrial content, size and number following demyelination in models and MS

Model	Mitochondrial content (% of axon area)	Mitochondrial size (μm^2^)	Mitochondrial number/10^3^ μm^2^ of axon area
Focal LPC	28.78 ± 9.98***	5.28 ± 1.77**	42.07 ± 19.39
	18.69 ± 3.98	2.93 ± 1.56	32.28 ± 19.36
Focal LPS	23.88 ± 12.98***	4.55 ± 2.24	56.40 ± 25.57*
	13.17 ± 3.73	4.58 ± 1.30	40.11 ± 14.02
Cuprizone‐mediated	23.01 ± 4.99***	4.75 ± 1.71**	68.81 ± 12.21***
	15.02 ± 3.57	2.92 ± 1.03	47.66 ± 7.71
TMEV‐induced	20.39 ± 2.88**	6.61 ± 2.29**	34.71 ± 8.62
	15.17 ± 2.04	4.56 ± 1.29	33.45 ± 10.92
TCR tg EAE	16.76 ± 7.30*	5.01 ± 1.09	40.76 ± 20.51*
	10.15 ± 4.00	4.26 ± 1.21	23.30 ± 13.13
EAE Biozzi ABH	20.81 ± 7.42***	4.93 ± 1.29	43.04 ± 14.74**
	12.79 ± 3.22	4.69 ± 1.24	27.75 ± 5.16
T‐reg depleted EAE	23.18 ± 9.79***	2.57 ± 0.97	53.46 ± 14.77
	15.38 ± 4.88	1.98 ± 1.42	32.04 ± 16.96
EAE rat	25.74 ± 7.72***	5.10 ± 2.62	54.90 ± 13.78***
	13.37 ± 3.91	3.78 ± 1.24	37.15 ± 10.39
EAE marmoset	19.51 ± 5.92*	3.08 ± 0.64	64.93 ± 21.44
	14.92 ± 8.33	3.31 ± 1.93	53.18 ± 12.49
Progressive MS [10]	19.61 ± 5.67**	15.67 ± 3.68**	49.21 ± 11.93
	6.26 ± 1.73	5.22 ± 1.92	33.60 ± 9.67

*Note*: Mitochondrial content (column two) is expressed as a percentage of axonal area occupied by porin‐labelled elements. Mitochondrial size (column three) and mitochondrial number (column four) are based on the area and number, respectively, of porin‐labelled elements within axons in confocal images. Shaded rows indicate mean values for myelinated axons from controls and unshaded rows mean indicate values for demyelinated axons. EAE: experimental autoimmune encephalomyelitis. LPC: lysolecithin. LPS: lipopolysaccharide. TCR tg: T‐cell receptor transgenic. TMEV: Theiler's murine encephalomyelitis virus. Values indicate mean ± standard deviation.

* *p* < 0.05

** *p* < 0.01 and

*** *p* < 0.001.

**TABLE 3:** Complex IV activity and complex IV subunit‐I relative to axonal area and complex IV subunit‐I relative to complex II subunit labelled area in myelinated axons and demyelinated axons

Model	Complex IV activity in all axonal mitochondria (% of axon area)	Complex IV subunit‐I in complex IV‐deficient mitochondria (% of complex II 70KDa area)	Complex IV subunit‐I in all axonal mitochondria (% of axonal area)
Focal LPC	12.81 ± 3.69**	85.49 ± 16.96	24.15 ± 8.37**
	7.44 ± 4.57	62.22 ± 11.59	10.25 ± 2.43
Focal LPS	6.08 ± 2.55	63.16 ± 21.29	15.51 ± 8.43**
	5.84 ± 1.78	69.13 ± 14.51	8.49 ± 2.48
Cuprizone‐mediated	10.50 ± 6.55	68.73 ± 14.24	17.30 ± 3.75**
	6.83 ± 1.55	61.34 ± 11.98	11.08 ± 2.25
TMEV‐induced	9.42 ± 6.37	58.41 ± 14.14	15.89 ± 2.91**
	4.98 ± 1.19	62.88 ± 10.87	10.89 ± 2.44
EAE, TCR tg	4.28 ± 2.48	73.46 ± 15.32	11.61 ± 5.31*
	4.59 ± 1.38	67.25 ± 11.14	7.72 ± 2.87
EAE, Biozzi ABH	3.69 ± 2.15	62.36 ± 21.20	15.16 ± 5.28**
	5.07 ± 2.16	67.54 ± 10.26	8.76 ± 2.09
EAE, T‐reg depleted	2.18 ± 1.15	64.73 ± 18.56	14.24 ± 4.96*
	6.10 ± 1.11	71.96 ± 11.90	9.32 ± 2.23
EAE, rat	4.36 ± 1.90	64.78 ± 20.50	17.59 ± 5.28**
	5.57 ± 2.50	67.83 ± 12.17	8.12 ± 2.38
EAE, marmoset	5.01 ± 1.39	66.49 ± 22.95	14.71 ± 4.46*
	5.74 ± 1.98	70.76 ± 18.49	10.13 ± 5.66
Progressive MS	7.37 ± 4.98** (Mahad et al, 2009)	22.82 ± 21.34**	10.10 ± 3.89**
	2.23 ± 1.62	51.89 ± 22.86	4.91 ± 3.05

*Note*: Mitochondrial respiratory chain complex IV active mitochondria in axons (column two) are assessed as a percentage of axonal area occupied by these complex IV active mitochondria. Complex IV subunit‐I is assessed in all axonal mitochondria as the percentage area of the subunit present within the axons (column three) in triple labelled images. When axonal mitochondria that lack complex IV activity are detected using complex II 70 kDa labelled elements within axons by the sequential complex IV histochemistry and triple labelling technique, the percentage area of complex IV subunit‐I labelling in the mitochondria are not significantly different between myelinated axons in controls and demyelinated axons in all the disease models (last columns). Shaded rows indicate values for myelinated axons and unshaded rows indicate values for demyelinated axons. EAE: experimental autoimmune encephalomyelitis. LPC: lysolecithin. LPS: lipopolysaccharide. TCR tg: T‐cell receptor transgenic. TMEV: Theiler's murine encephalomyelitis virus. Values indicate mean ± standard deviation.

* *p* < 0.01 and

** *p* < 0.001.

#### Triple immunofluorescence histochemistry following complex IV histochemistry

To assess the complex IV activity in axons, a mask of the individual axon identified by the fluorescent labelling of NF‐H was generated on Image J and superimposed onto the brightfield image of complex IV histochemistry. The total area occupied by complex IV active elements within a single axon was determined as a percentage of the axonal area; 20 axons (at least 25 μm in length) per region were randomly selected from each animal for quantitation and the mean values are indicated in Table 3. SCoRe analysis by reflecting the signal from multiple layers of available wavelengths was used to ensure confirmation of myelinated and demyelinated axons. For the localisation of axonal mitochondria that lack complex IV activity and the quantitation of their complex IV subunit‐I, immunofluorescent images of the complex IV‐deficient mitochondria labelled with complex II 70 kDa subunit were obtained using the Apotome. Once the non‐axonal mitochondria that were outside the axon outline were removed from the Z‐stack, a mask of complex II 70 kDa labelled axonal elements was obtained and then combined with the mask of complex IV subunit‐I labelled elements. The extent of axonal complex IV subunit‐I within the complex IV‐deficient mitochondria labelled with complex II 70KDa was then calculated, as a percentage, by dividing the sum of the area of complex IV subunit‐I elements by the sum of the complex II 70 kDa labelled elements in a given axon (Table 3, columns four); 20 axons per region were randomly selected from each animal for quantitation and the mean values are indicated in Table 3. Assessors were blinded by coding the axons in ascending numerical order.

#### APP and synaptophysin

Quantification was performed on adjacent sections with immunofluorescent histochemistry for APP or synaptophysin and NF‐H, following complex IV histochemistry. Inflammatory demyelinated lesions were identified based on hypercellular regions as observed by DAPI and serial sections of tissue, which showed loss of MBP. Areas within the lesion were randomly chosen and the number of APP and synaptophysin reactive elements were counted using Image J software. The density of APP and synaptophysin labelled elements per mm^2^ was therefore determined.

### Statistics

Mann–Whitney U tests using GraphPad Prism 6 were carried out to compare mitochondrial occupancy and complex IV activity in myelinated and demyelinated axons for each model. Where multiple data sets were compared between models, the Kruskal–Wallis test was carried out. To identify significant associations, a Spearman correlation test was carried out in GraphPad Prism. A *p* value of <0.05 was considered statistically significant.

## RESULTS

### Increased mitochondrial content within demyelinated axons indicates ARMD in experimental disease models irrespective of the mode of demyelination

**FIGURE 1:**
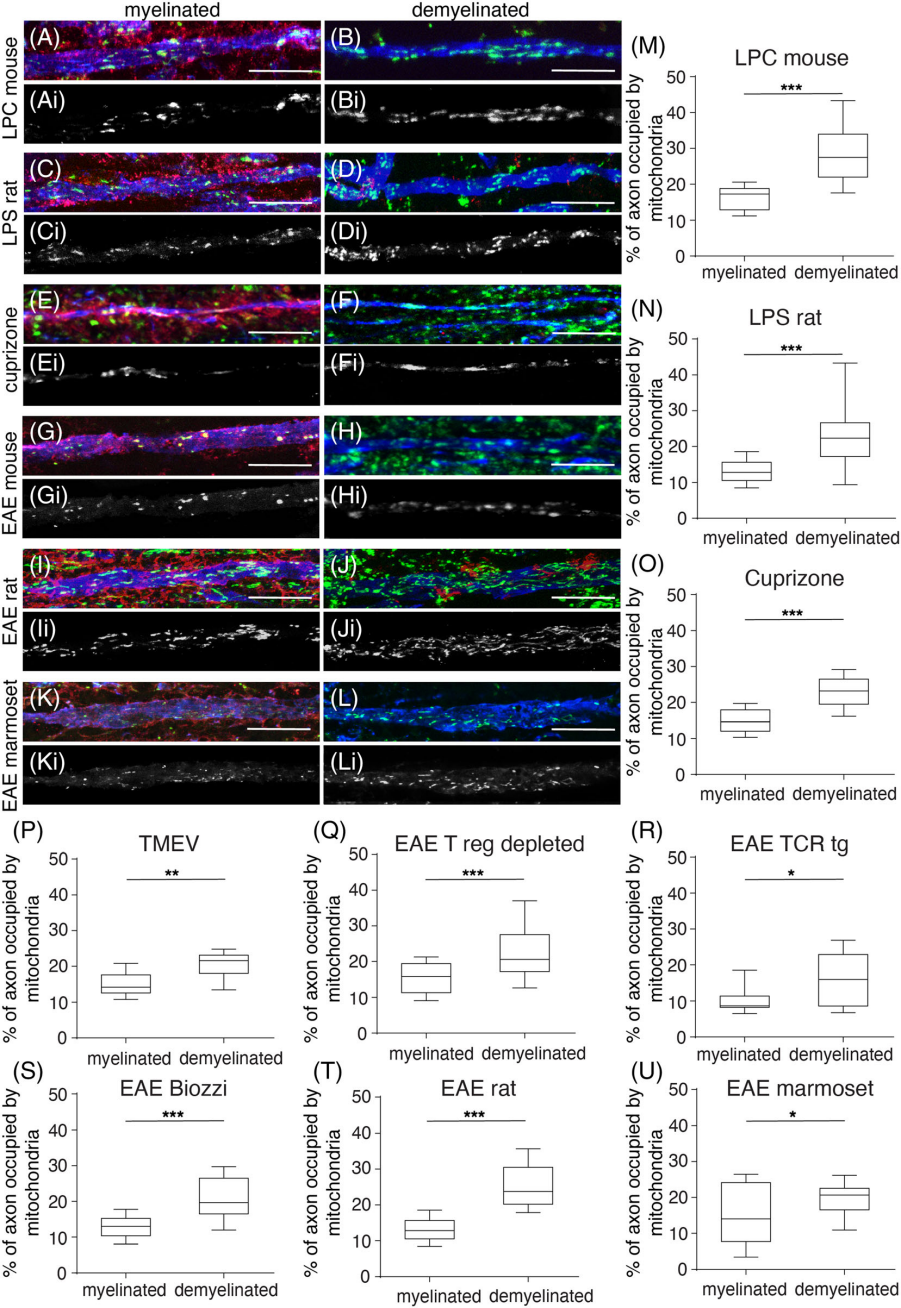
Axonal mitochondrial content consistently increases upon experimental demyelination. Compared with myelinated axons from controls, in triple labelled immunofluorescent confocal images (column to the left with MBP in red, neurofilament‐H in blue and porin in green), mitochondria are more prevalent in demyelinated axons (column to the right) from all the models. The greyscale images (Ai‐li) show porin‐positive elements within axons from the corresponding triple labelled colour images (A‐L). The quantitation of axonal mitochondrial content shows a significant increase in the lysolecithin‐induced focal lesions (LPC, A‐B and M), lipopolysaccharide‐induced focal lesions (LPS, C‐D and N), cuprizone model (E‐F and O), Theiler's murine encephalomyelitis virus (TMEV) model (P) as well as experimental autoimmune encephalitis (EAE, G‐L and Q‐U) in mice (C57BL6, SJL/J and Biozzi ABH), rat (dark agouti) and marmoset species (the area of porin‐positive elements as a percentage of axon area); 20 axons per region were randomly selected from each animal for quantitation. The box plots indicate the median, inter‐quartile range (25%–75%) and 90% confidence interval. *p < 0.05, **p < 0.01 and ***p < 0.01. The scale bar indicates 10 μm.

We assessed the mitochondrial content of demyelinated axons in nine different disease models at peak clinical disease or, when a clinical phenotype is not yet present, peak demyelination time point (Table 1) and compared it with myelinated axons in controls (Table 2). Mitochondria within demyelinated axons, selected by neurofilament labelling and lack of myelin basic protein (MBP) immunofluorescence, were identified in confocal images based on immunofluorescent labelling of porin, which is a voltage‐gated anion channel (VDAC) expressed in all mitochondria. (Figure 1). The mitochondrial content within demyelinated axons was significantly increased in the spinal cord of all EAE, lysolecithin, lipopolysaccharide (LPS) and Theiler's murine encephalomyelitis virus (TMEV)‐induced models as well as in the corpus callosum in cuprizone mediated demyelination, compared with myelinated axons in controls, indicating the presence of ARMD (Figure 1 and Table 1). The significant increase in mitochondrial content within demyelinated axons of all models arose from increased mitochondrial size and/or increased mitochondrial number (Table 2). In lysolecithin and cuprizone‐mediated demyelination and TMEV‐induced demyelination, the average axonal mitochondrial size was significantly greater than in control myelinated axons. Meanwhile, the greater mitochondrial number accounted for the increased mitochondrial content in EAE models. To assess whether the myelinated segments of peri‐lesional axons also contain increased mitochondrial content, we analysed peri‐lesional spinal cord white matter in lysolecithin‐induced focal demyelination. Myelinated axons in peri‐lesional white matter contained significantly fewer mitochondria (9.96 ± 2.74, *n* = 7 mice) than demyelinated axons (28.78 ± 9.98, *p* < 0.0001) as well as myelinated axons in control mice (18.69 ± 3.98, *p* = 0.0002). These observations show that ARMD is a consistent phenomenon irrespective of the mode of demyelination.

### An increase in axonal mitochondrial content is not always accompanied by a corresponding increase in mitochondrial respiratory chain complex IV activity

**FIGURE 2:**
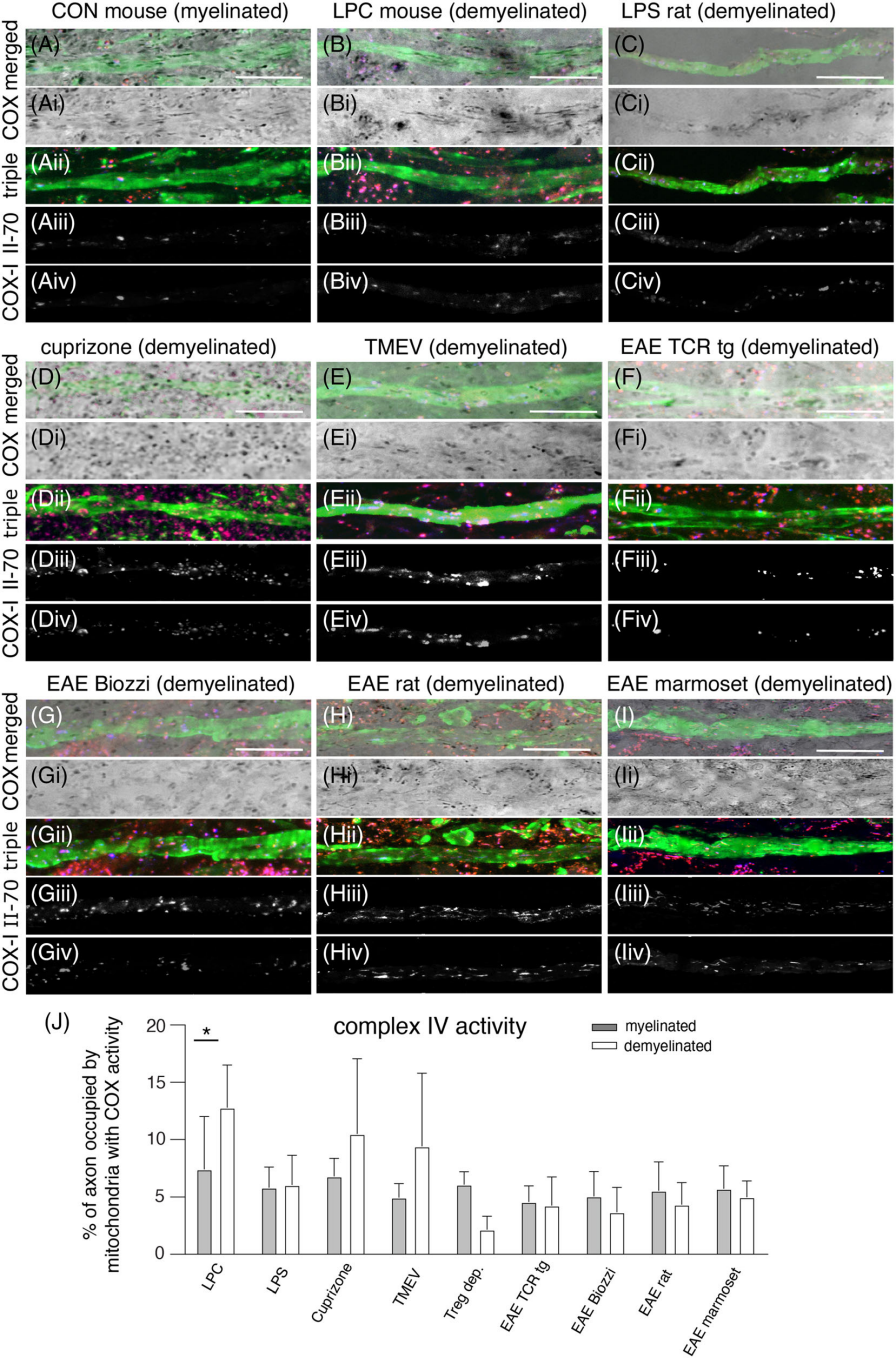
Complex IV activity within axons and the detection of complex IV subunit‐I relative to complex II 70 kDa in complex IV‐deficient axonal mitochondria. Complex IV activity can be localised to the axon by the sequential complex IV histochemistry (bright field images, Ai–Ii) and triple immunofluorescent labelling (Aii–Iii) of neurofilament (green), complex II 70 kDa subunit (red) and complex IV subunit‐I (blue) and then by merging the bright field image with triple labelled immunofluorescent image (A–I). This sequential technique immunofluorescently labels the complex IV‐deficient mitochondria (labelled with complex II 70 kDa, Aiii–Iiii) and their mitochondrial respiratory chain complex subunits (Aiv‐Iiv), as previously described [16]. The grey scale immunofluorescent images of axonal complex II 70 kDa (Aiii–Iiii) and complex IV subunit‐I (Aiv–Iiv) are generated by splitting the corresponding triple labelled colour image (Aii–Iii) and clearing the non‐axonal mitochondria. As reported previously, the mitochondria with complex IV activity, evident in the bright field images, are not immunofluorescently labelled [16]. Following lysolecithin‐induced (LPC) demyelination (panel B), there are numerous axonal mitochondria with complex IV activity and elongated morphology (B and Bi) compared with myelinated axons from controls (A and Ai). In contrast, mitochondria with complex IV activity in demyelinated axons are less numerous and rounded or less elongated in all other models [lipopolysaccharide‐induced (LPS) lesions (C,Ci), cuprizone model (D,Di), TMEV‐induced model and experimental autoimmune encephalitis (EAE) in mouse, rat and marmoset]. The quantitation of complex IV activity within axons shows a significant increase following LPC‐induced focal demyelination (J); 20 axons per region were randomly selected from each animal for quantitation. The bar charts indicate the mean plus standard deviation. *p < 0.001. The scale bar indicates 10 μm.

To determine whether the increased mitochondrial content within demyelinated axons is reflected at the functional level, we assessed complex IV activity of mitochondria at a single axon level using an established technique involving sequential complex IV histochemistry and immunofluorescent labelling of axons in snap frozen serial cryosections (Figure 2) [16]. This sequential technique labels mitochondria with complex IV activity (in brightfield image). Furthermore, this technique identifies mitochondria that lack complex IV activity (in immunofluorescent images) and permits the determination of the subunit status of mitochondria that lack complex IV activity, as previously described [9, 16]. Quantification of complex IV activity within axons revealed a significantly greater area of the demyelinated axons occupied by complex IV active mitochondria in lysolecithin‐induced lesions (Figure 2 and Table 3). In the lysolecithin‐induced focal lesions, complex IV active mitochondria showed an elongated morphology and reflected the increased mitochondrial content of demyelinated axons (Figure 2).

In highly inflammatory models, such as EAE, we did not find a significant increase in complex IV activity within demyelinated axons (Table 3), despite the increase in axonal mitochondrial content upon demyelination (Table 2). Although complex IV activity had a tendency to increase within demyelinated axons in cuprizone and TMEV models the difference was not statistically significant. As the relative lack of complex IV activity within demyelinated axons, particularly in highly inflammatory models may be due to the loss of complex IV subunits, we proceeded to assess complex IV subunit‐I using triple immunofluorescence histochemistry following complex IV histochemistry.

### Mitochondrial respiratory chain complex IV subunit‐I is preserved in complex IV deficient axonal mitochondria in all experimental disease models

In axonal mitochondria that lack complex IV activity, the extent of complex IV subunit‐I (COX‐I) labelling was similar in demyelinated axons when compared with myelinated axons, suggesting that the lack of complex IV activity is not caused by the loss of complex IV subunit‐I. To confirm that the complex IV subunit is intact in demyelinated axons, we immunofluorescently co‐labelled mitochondria and the subunit in serial sections and found a significant increase in complex IV subunit‐I within demyelinated axons in all models, reflecting the increased mitochondrial content, compared with myelinated axons (Table 3).

### Mitochondrial complex IV activity in demyelinated axons inversely correlates with axonal injury in experimental disease models

**FIGURE 3:**
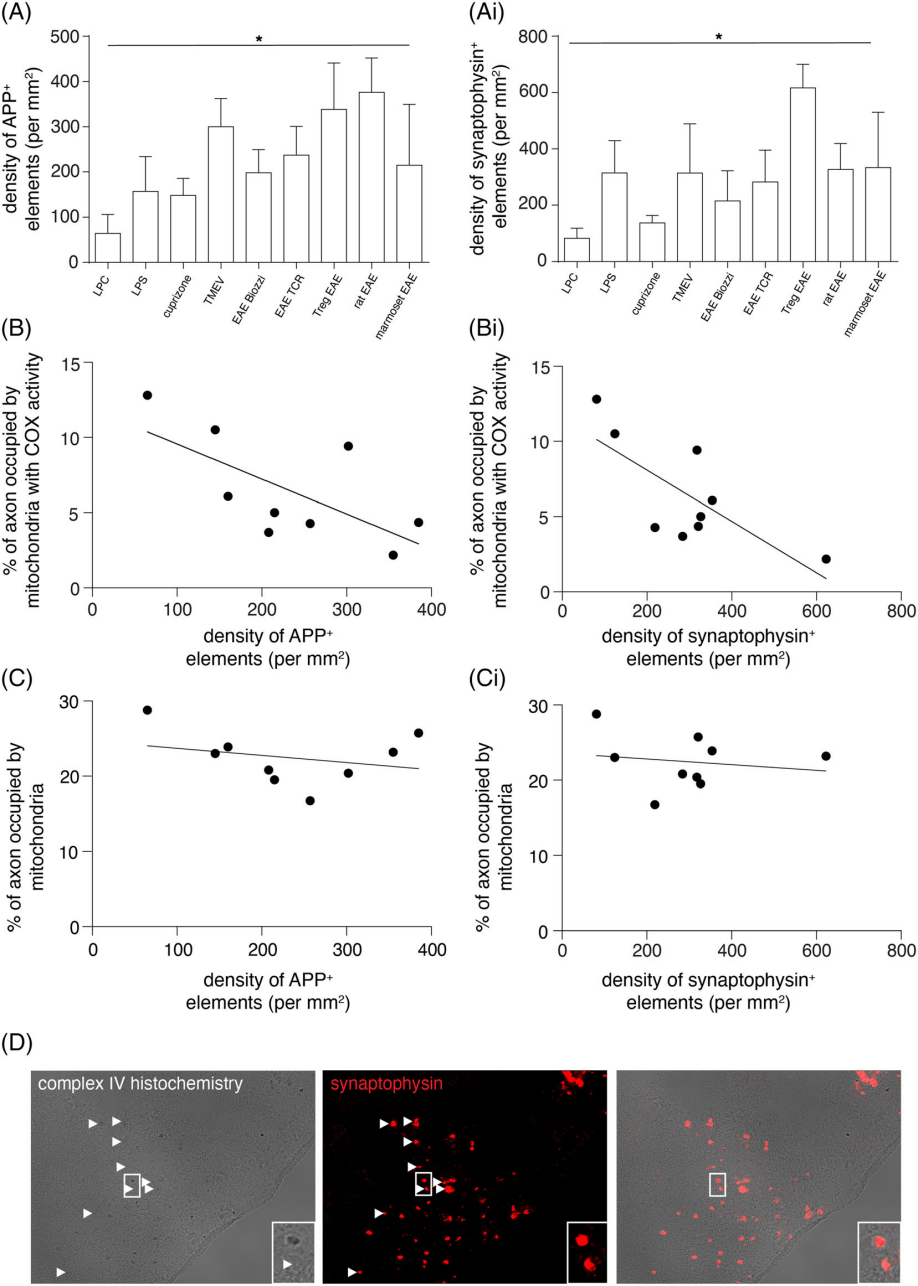
The association between complex IV activity within demyelinated axons and the extent of axonal injury. The density of axonal injury, judged by amyloid precursor protein (APP, A) and synaptophysin (Ai) labelling, varies considerably between the disease models (Kruskal–Wallis *p* < 0.001). There is a significant inverse correlation between complex IV activity within demyelinated axons and axon degeneration, judged by the density of APP (B, *r* ^2^ = 0.421, *p* = 0.048) as well as synaptophysin (Bi, *r* ^2^ = 0.561, *p* = 0.020) labelling. In contrast, a significant correlation is not found between mitochondrial content in demyelinated axons and the density of APP (C, *r* ^2^ = 0.040, *p* = 0.604) and synaptophysin (Ci, *r* ^2^ = 0.147, *p* = 0.308) labelling. Sequential complex IV histochemistry and immunofluorescent labelling of APP shows that a subset of APP and synaptophysin labelled structures contains mitochondria with complex IV activity in all nine disease models (D, synaptophysin positive structures lacking complex IV activity are shown in T‐reg depleted EAE lesion, arrowheads). Each data point in B–Bi and C–Ci indicates the mean value of a single model. From left to right in B and C, data points are in the following order: LPC, cuprizone, LPS, EAE biozzi, marmoset EAE, EAE TCR, TMEV, Treg EAE and rat EAE. From left to right in Bi and Ci, data points are in the following order: LPC, cuprizone, EAE TCR, EAE biozzi, TMEV, rat EAE, marmoset EAE, LPS and Treg EAE. The bar charts indicate the mean plus standard deviation.

To assess the relationship between our findings and axonal damage, we correlated axonal mitochondrial parameters (content and complex IV activity) with the extent of axonal injury in all nine models, as indicated by the density of APP and synaptophysin positive elements (Figure 3). The greatest extent of axonal injury was observed in highly inflammatory models, such as EAE, whereas lysolecithin induced lesions contained the least extent of axonal injury (Figure 3A‐Ai). At the level of complex IV activity, there was a significant inverse correlation between the mean complex IV activity within demyelinated axons and the density of APP‐positive elements (Figure 3B). Synaptophysin positive elements also showed a significant inverse relationship with axonal complex IV activity (Figure 3Bi). However, not all APP‐positive and synaptophysin‐positive elements were complex IV deficient (Figure 3D). In contrast to complex IV activity, we did not detect a significant correlation between mitochondrial content of demyelinated axons and axonal damage (Figure 3C,Ci).

## DISCUSSION

We recently reported a homeostatic mechanism in neurons, termed ARMD, where mitochondria move from the cell body to the axon upon lysolecithin‐induced focal demyelination [5]. In the present study, we show that ARMD is a consistent feature of demyelinated axons in nine experimental disease models. Additionally, we assessed axonal complex IV activity and found that the increased axonal mitochondrial content in demyelinated axons is not always reflected by an increase in complex IV activity, particularly in the highly inflammatory EAE models studied. Furthermore, axonal complex IV activity inversely correlated with axonal injury. These findings indicate that complex IV activity in the context of ARMD is likely to be important for axonal health, especially in inflammatory demyelinating environments.

Previous studies have identified increased mitochondrial content in demyelinated axons in ethidium bromide (EB) lesions, demyelinated cat optic nerve and Theiler's murine encephalomyelitis virus (TMEV) as well as in MS [6, 7, 9, 10]. In this study, we show that the axonal mitochondrial content consistently increases, irrespective of the mode of demyelination, due to increased size and/or number of axonal mitochondria. The lack of increased mitochondrial size in inflammatory demyelination may be due to mitochondrial fragmentation, as previously reported in EAE, as well as decreased fusion of mitochondria in demyelinated axons. Whether mitochondrial fusion occurs within demyelinated axons in EAE, before mitochondrial fragmentation, needs to be investigated using live imaging techniques. While the increased axonal mitochondrial content has been proposed as a pathogenic mechanism, a study that prevented the increase of mitochondrial content in demyelinated axons by disrupting mitochondrial docking established that ARMD is a homeostatic and protective mechanism [8]. Furthermore, recent studies that enhanced ARMD, by over‐expressing PGC1α in neurons as well as pharmacologically targeting PGC1α to increase mitochondrial biogenesis, showed protection of acutely demyelinated axons in EAE and lysolecithin‐induced lesions [5, 18]. Our current findings in nine experimental models establish that homeostatic ARMD is a consistent phenomenon, irrespective of the mode of demyelination.

**FIGURE 4:**
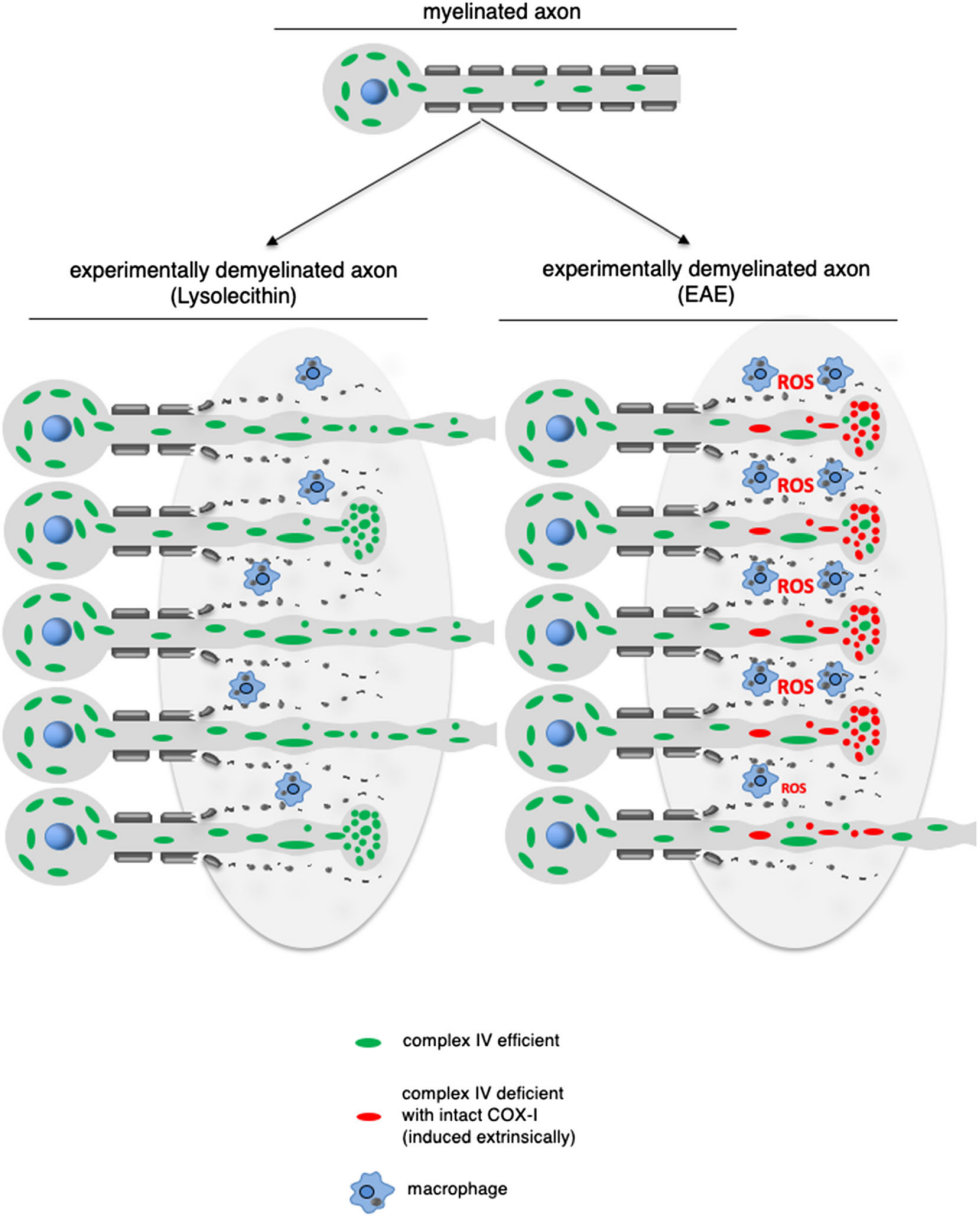
The role of complex IV in ARMD. Consistent with ARMD, demyelinated axons show increased mitochondrial content compared with myelinated axons (green and red mitochondria), irrespective of the mode of experimental demyelination (as depicted for the lysolecithin model and EAE). At the level of complex IV, lysolecithin‐induced lesions show a significant increase in mitochondria with complex IV activity (green mitochondria) compared with myelinated axons, reflecting the increased axonal mitochondrial content. In contrast, the increased mitochondrial content of demyelinated axons in EAE is not accompanied by a corresponding increase in complex IV activity. Instead, demyelinating axons in EAE are abundant in complex IV deficient mitochondria (red). Intact COX‐I in complex IV deficient axonal mitochondria suggests that the complex IV deficiency is due to inhibition by nitric oxide and subunit modification by reactive oxygen species (ROS). The lack of complex IV activity in demyelinated axons in EAE, despite the increased mitochondrial content, is associated with a substantially greater extent of axon injury (axonal ovoids). This raises the possibility that complex IV is important for the health of acutely demyelinated axons.

Whether the increased mitochondrial content of demyelinated axons is reflected at the level of mitochondrial complex IV activity had not been studied in experimental disease models. In this study, we show that complex IV activity of demyelinated axons is dependent on the mode of experimental demyelination (Figure 4). In lysolecithin‐induced lesions, the significant increase in complex IV activity within demyelinated axons is comparable to the extent of complex IV activity within dysmyelinated axons in *shiverer* mice, where autoimmune inflammation is also not modelled [19]. In contrast, autoimmune inflammation (EAE) did not lead to a significant increase in axonal complex IV activity despite the increase in axonal mitochondrial content (Figure 4). The lack of complex IV activity in axonal mitochondria that have responded to demyelination in EAE may be due to a number of potential molecular mechanisms. First, a previous study has shown that modification of complex IV subunits occurs in EAE, although this was not localised to particular cellular structures [12]. Second, an excess of nitric oxide which is known to compete with oxygen and inhibit complex IV is evidenced by the increase in iNOS in acute EAE [5, 13] Nitric oxide is also implicated in axon degeneration due to the correlation between iNOS expression and acute axonal injury [20]. Based on the relatively low expression of iNOS in LPS lesions and cuprizone model, relative to EAE that we previously reported, inhibition of complex IV by nitric oxide is likely to be only one of several molecular mechanisms of axonal complex IV deficiency [3, 5, 21]. In TMEV‐induced demyelination, the relative sparing of complex IV activity at 41 days may be due to weak expression of iNOS and relative lack of reactive oxygen species at this stage, as previously reported [22, 23]. Third, reactive oxygen species damage axonal mitochondria and disrupt axonal mitochondrial transport in EAE [15, 24]. The differential complex IV activity within axons in toxic demyelination and autoimmune inflammatory demyelination raises the possibility that inflammation damages complex IV in demyelinating models.

Our findings of intact COX‐I in axons indicate that the lack of complex IV activity in experimentally demyelinated axons in EAE is not due to mitochondrial DNA (mtDNA) mutations or loss of transcripts. These observations in EAE are supported by previous studies of transcripts of mitochondrial respiratory chain subunits and mtDNA, both of which were unaltered in experimental models [5, 25]. A limitation of this study is that all the experimental disease models that represent complex IV deficiency in axons, at a mechanistic level are only due to inflammation‐induced complex IV deficiency, rather than the irreversible complex IV loss due to mitochondrial DNA mutations that are found in MS [5, 26].

Complex IV activity is important for axonal health in non‐demyelinating disorders and is likely to be even more relevant in demyelinating disorders as a result of the increased energy demand of demyelinated axons. For the first time, we show an inverse relationship between complex IV activity in demyelinated axons and the extent of axon degeneration in experimental disease models, raising the possibility that complex IV is important for the health of acutely demyelinated axons. We recently showed that enhancing ARMD in complex IV deficient neurons can protect demyelinated axons in COX10Adv mice, where complex IV subunit 10 in dorsal root ganglia neurons is inducibly knocked out [5]. This indicates that complex IV deficiency in axons plays a role in axon degeneration through the loss of function or lack of energy rather than due to a toxic gain of function. Unlike in COX10Adv mice, where the complex IV deficiency and the loss of subunits are irreversible, complex IV deficiency due to inhibition, for example, by nitric oxide, and modification of subunits by reactive oxygen species is potentially reversible. This reversibility stems from the ability of neurons in experimental disease models to generate healthy mitochondria as they do not show mtDNA mutations or loss of nuclear DNA encoded transcripts [5, 25]. In these experimental disease models, newly generated mitochondria and their movement from the cell body to the axon may replace damaged mitochondria and restore axonal energy production. In MS, complex IV deficiency of axons is caused by multiple mechanisms, including nitric oxide‐mediated inhibition of complex IV, inflammation‐related direct damage to complex IV as well as the chronic nature of oxidative injury leading to mtDNA deletions. Besides complex IV deficiency, other mechanisms lead to axon degeneration in the context of demyelination, as reflected by the presence of complex IV activity in a subset of degenerating axons in our animal models. However, the occurrence of potentially reversible complex IV deficiency due to inflammation‐induced mitochondrial damage, alongside the irreversible complex IV deficiency due to mitochondrial DNA mutations in MS, offers therapeutic potential [9, 26].

## CONCLUSIONS

In summary, we show that ARMD is a consistent feature of a wide range of experimental disease models and highlight the importance of complex IV activity for the health of acutely demyelinated axons. Our findings suggest that the enhancement of ARMD, which we reported as a neuroprotective strategy for MS, may be further optimised by limiting complex IV deficiency, especially in highly inflammatory environments.

## CONFLICTS OF INTEREST

DM, GC, SLM and SC are inventors on patent EI0000273 named ‘enhancement of ARMD response as therapy in multiple sclerosis’.

## AUTHOR CONTRIBUTIONS

SLM and GRC contributed to tissue staining, imaging, acquisition, analysis and interpretation of data as well as drafted the manuscript. ARM helped with data interpretation and substantially revised the manuscript. KM performed immunostaining and cryosectioning of tissue. AS contributed to the quantification of mitochondrial parameters. SA, GP, SZ, CZ, MK, KJS, DB, DA, SMA, YSK, JDL, BAtH, MR and RJMF contributed to the generation of animal models and study design. SC, HL and BDT contributed to data interpretation and substantial revision of the manuscript. DJM contributed to the conception and design of the study, data interpretation and drafting of the manuscript.

## ETHICS STATEMENT

All animal experiments performed in the United Kingdom complied with the Animals (scientific procedures) Act 1986 and UK Home Office guidelines under the animal licence (PPL 70/7872). Tissue from established animal models was obtained through collaborations, as listed in Table 2, and complied with local animal research and ethics rules.

## CONSENT FOR PUBLICATION

Not applicable.

### PEER REVIEW

The peer review history for this article is available at https://publons.com/publon/10.1111/nan.12851.

## Data Availability

All data generated or analysed during this study are included in the published article in graphic format and tabulated.
